# Somatic Alpha-Synuclein Mutations in Parkinson's Disease: Hypothesis and Preliminary Data

**DOI:** 10.1002/mds.25502

**Published:** 2013-05-14

**Authors:** Christos Proukakis, Henry Houlden, Anthony H Schapira

**Affiliations:** 1 Department of Clinical Neuroscience, Institute of Neurology, University College LondonLondon, United Kingdom; 2 Department of Molecular Neuroscience, Institute of Neurology, University College LondonLondon, United Kingdom

**Keywords:** SNCA, alpha-synuclein, somatic mutation, mosaicism, etiology of Parkinson's disease

## Abstract

Alpha-synuclein (SNCA) is crucial in the pathogenesis of Parkinson's disease (PD), yet mutations in the *SNCA* gene are rare. Evidence for somatic genetic variation in normal humans, also involving the brain, is increasing, but its role in disease is unknown. Somatic *SNCA* mutations, arising in early development and leading to mosaicism, could contribute to PD pathogenesis and yet be absent or undetectable in DNA derived from peripheral lymphocytes. Such mutations could underlie the widespread pathology in PD, with the precise clinical outcome dependent on their type and the timing and location of their occurrence. We recently reported a novel *SNCA* mutation (c.150T>G, p.H50Q) in PD brain-derived DNA. To determine if there was mosaicism for this, a PCR and cloning strategy was used to take advantage of a nearby heterozygous intronic polymorphism. No evidence of mosaicism was found. High-resolution melting curve analysis of *SNCA* coding exons, which was shown to be sensitive enough to detect low proportions of 2 known mutations, did not reveal any further mutations in DNA from 28 PD brain-derived samples. We outline the grounds that make the somatic *SNCA* mutation hypothesis consistent with genetic, embryological, and pathological data. Further studies of brain-derived DNA are warranted and should include DNA from multiple regions and methods for detecting other types of genomic variation. © 2013 *Movement* Disorder Society

Alpha-synuclein (SNCA), encoded by the *SNCA (PARK1)* gene, is central to the pathogenesis of Parkinson's disease (PD).^1^,^2^ It is the major component of Lewy bodies. Misfolding into oligomers and fibrils is believed to underlie its toxicity, although the precise nature of the toxic species remains unclear. Three *SNCA* missense mutations have been reported in pedigrees with autosomal dominant inheritance.^3^–^5^ We recently identified the c.150T>G/p.H50Q mutation in DNA derived from the brain of a single apparently sporadic case of late-onset PD.^6^ Copy number variations (CNVs, duplications and triplications) have also been described,^7^,^8^ and noncoding variation in *SNCA* is a risk factor for sporadic PD.^9^ Several large studies analyzing DNA from blood lymphocytes have not found additional mutations, including 1 of more than 1900 mostly sporadic patients.^10^

Mutations occurring postzygotically are termed somatic and can lead to mosaicism (the presence of more than 1 genetically distinct cell in a single organism).^11^ The number of cell divisions in normal development has led to the suggestion that each gene may mutate several times postzygotically, with the term *somatic evolutionary genomics* used to refer to the accumulation of genetic change within the cell lineage of a single individual.^12^ Somatic mutations occurring in early embryogenesis in a dividing cell whose progeny will include neurons, derived from the ectoderm, could contribute to PD, but they could be missed when mesoderm-derived lymphocyte DNA is analyzed, in which they might be absent or present at a level below the 15%–30% resolution limit of Sanger sequencing.^13^–^15^ Low-level mosaicism could have been missed even in the few small studies in which *SNCA* was analyzed in PD brain-derived DNA, as the methods were not sensitive enough.^16^–^18^ Somatic mutation has previously been suggested as a cause of sporadic neurodegenerative disorders^19^,^20^ and was very recently hypothesized as a possible explanation of phenotypically discordant LRRK2 monozygotic twins.^21^,^22^ In Alzheimer's disease, a case with mosaicism from somatic mutation of presenilin-1 was described, with 14% mutant DNA in the cortex.^23^ Hereditary spastic paraplegia caused by mosaicism for a spastin mutation has been reported.^24^ Very recently, a novel form of neurodegeneration with brain iron accumulation has been found to be a result of mutations in WDR45, with a somatic origin in some cases.^25^ Mosaicism for triplet-repeat neurodegenerative disorders from somatic mutation of the expanded repeat has been described,^26^,^27^ including fragile X premutation syndrome, in which somatic instability in brain appears more pronounced than in blood,^28^ and c9orf72 in amyotrophic lateral sclerosis.^29^ Mosaicism for the expanded repeat may be the cause of intrafamilial variation in Friedreich's ataxia^30^ and is associated with onset age in Huntington's disease.^31^ In the special case of the mitochondrial genome, heteroplasmic somatic DNA deletions and point mutations in the substantia nigra (SN) are associated with PD.^32^–^34^

We therefore decided to test the hypothesis that *SNCA* somatic coding mutations present in the brain may contribute to PD by investigating the possibility of mosaicism for the H50Q mutation, and by using high-resolution melting curve (HRM) analysis for detection of possible low-proportion mosaicism in PD brain-derived DNA.

## Patients and Methods

DNA from the brains of 28 patients with idiopathic PD from the Queen Square Brain Bank was analyzed. Patients had given informed consent for use of their brains in research, and the study was approved by the local ethics committee. The demographics and clinical detail of this cohort are summarized in Supporting Table 1. DNA was available from the SN and cerebellum in 5 cases, from the cerebellum in 7 cases, and from the caudate nucleus in 16 cases. The SN DNA had been previously sequenced in all 5 of the cases in which it was available, leading to the detection of the c.150T>G/p.H50Q mutation in 1 case.^6^ HRM analysis of PCR amplicons for all coding exons was performed using Idaho Technology HRM mastermix, amplicon melting on a Lightscanner (Idaho Technology, Salt Lake City, UT) and melt curves analysis with Call-IT 2.0 software. Further details and additional primers used for subcloning are shown in Supporting Table S1. HRM analysis is a robust and efficient method for screening small PCR amplicons for unknown mutations that relies on altered melting of heteroduplexes. Sensitivity is >99% for heterozygous point mutations and small insertions/deletions. In the case of mosaicism, a 5%–10% proportion of mutant DNA is detectable by HRM analysis,^15^ although detection of mutation proportion as low as 0.5% has been reported,^35^ making it highly suitable for screening for low-level mutations; the main limitation is the ability to analyze only small PCR amplicons, ideally 150–250 base pairs long.^15^

## Results

**FIG. 1:**
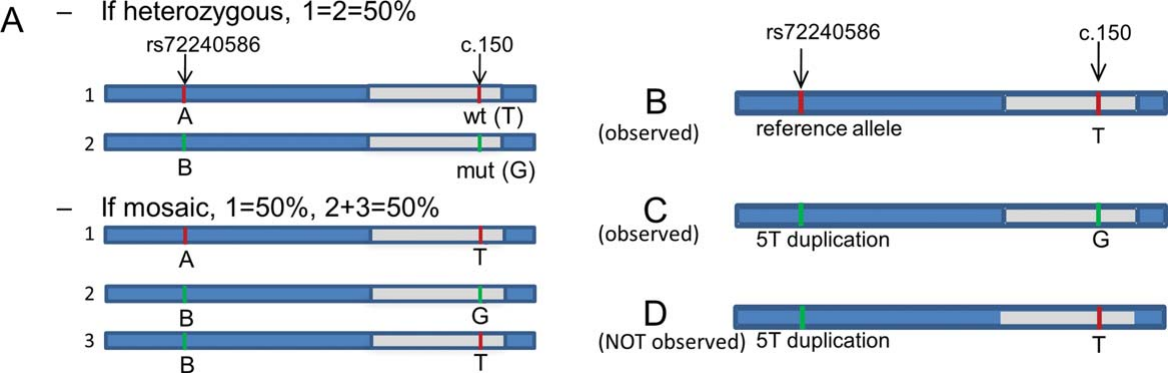
Determination of allele combinations at the rs72240586 polymorphic locus and c.150 (exon shown as light gray). **A:** Possible allele combinations in heterozygosity and mosaicism. The third combination indicates somatic origin of the c.150T>G mutation on the chromosome carrying rs72240586 allele B, with mosaicism evident, as both T and G are found in *cis* with allele B. **B:** Allele combination observed in chromosome without c.150T>G mutation. **C:** Allele combination observed in chromosome with c.150T>G mutation. **D:** Hypothetical third combination (*not* observed), which would have proven mosaicism by indicating that the mutation had arisen on the chromosome with the duplicated rs72240586 allele, but the original wild-type allele in *cis* with the rs72240586 duplication allele was also present.

Because of the a priori very low likelihood of an apparently sporadic late-onset PD case harboring a novel heterozygous missense mutation, we considered the possibility that the patient in whom we recently detected the novel c.150T>G (p.H50Q) mutation in the SN and cerebellum^6^ might have been a mosaic for a somatic mutation. Direct testing for the mutation in other tissues or in brain regions other than the SN or cerebellum was not possible, as no material was available. Inheritance could not be investigated, as relatives could not be traced. Mosaicism can be indirectly confirmed by determining the phase of a mutation in relation to a nearby heterozygous single-nucleotide polymorphism if a third allelic combination is present.^23^ Sequencing a PCR product including exon 3 but extending 430 bp upstream into intron 2 revealed heterozygosity for a known 5-base polymorphic insertion duplication (c.122–133_122–129dupTTTTT, rs72240586). A person heterozygous for both the c.150T>G exonic mutation and the 5T intronic duplication should have only 2 allele combinations, with the T and G at position c.150 each in *cis* with 1 of the rs72240586 variants; the presence of a third allele combination would indicate mosaicism and prove a somatic event (Fig. 1A). SN and cerebellar DNA was therefore amplified and subcloned in 2 independent experiments each; restriction digestion using *Bsg*I (for which c.150T>G generates a restriction site) of direct colony PCR products was used to differentiate wild-type (wt) and mutant colonies. Sequencing of mutant colony PCR products from the SN and cerebellum revealed c.150G to be in *cis* with the rs72240586 duplication allele (Fig. 1B), whereas sequencing of all wt colony PCR products from 1 experiment of each DNA source demonstrated c.150T to be *in cis* with the reference (nonduplicated) rs72240586 allele (Fig. 1C), with no colony PCR products showing c.150T in *cis* with the rs72240586 duplication allele (Fig. 1D). Therefore, there was no evidence of mosaicism.

**FIG. 2:**
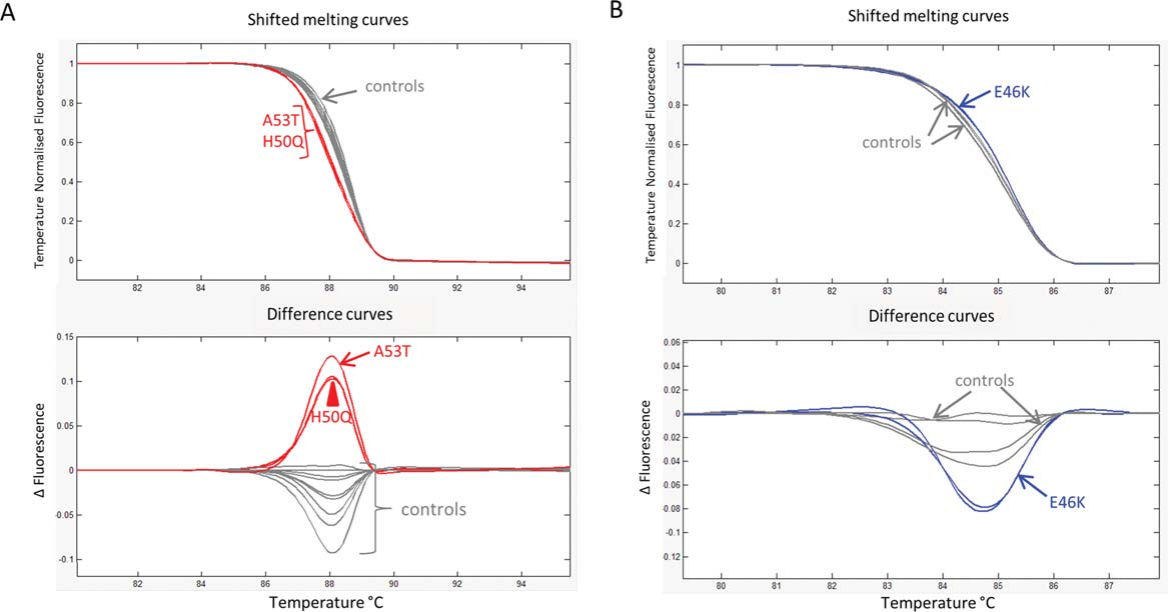
HRM analysis of all known exon 3 mutations. **A:** Heterozygote c.157G>A (p.A53T) and c.150T>G (p.H50Q) in duplicate are differentiated from 3 controls in triplicate. **B:** c.136G>A (p.E46K) heterozygote in duplicate is differentiated from 2 controls in duplicate.

**FIG. 3:**
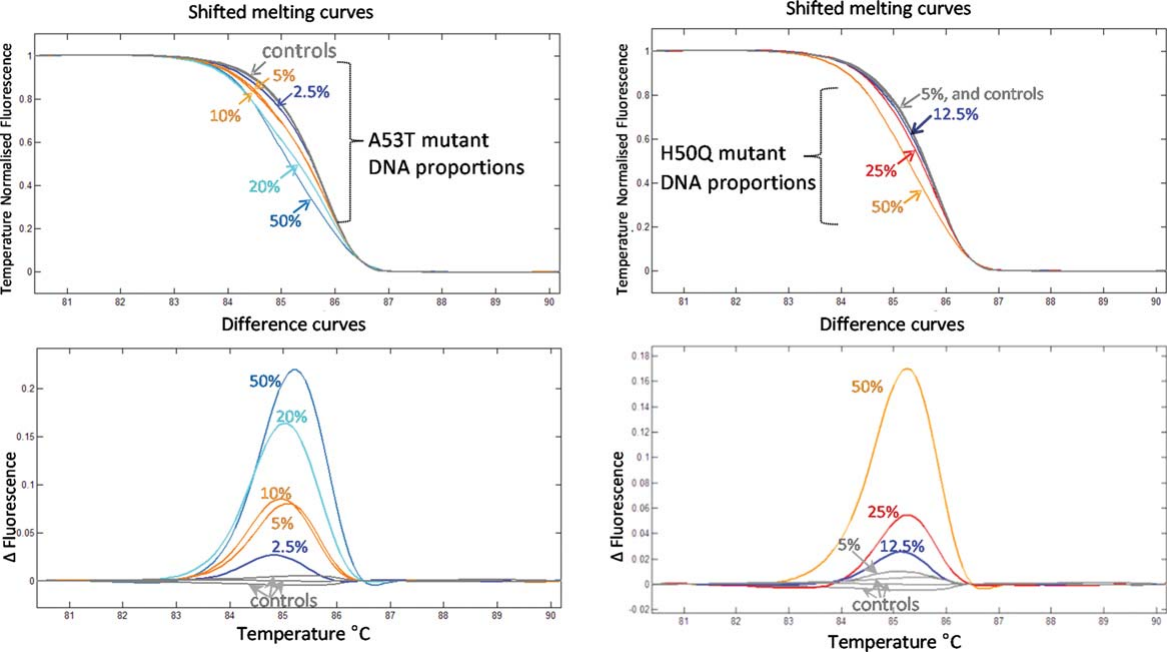
HRM analysis of DNA-carrying mutations, undiluted and diluted with control DNA, against control DNA in triplicate. The percentage of mutant DNA in each PCR template is shown. Left, c.157G>A (p.A53T), undiluted mutant percentage 50%, differentiated at 2.5% (1 in 20 dilution). Right, c.150T>G (p.H50Q), undiluted mutant percentage 50%, differentiated at 12.5% (1 in 4 dilution), not clearly differentiated at 5%.

To detect any *SNCA* somatic mutations that might be present at levels below the sensitivity of Sanger sequencing, we developed an HRM analysis protocol for the coding exons (2–6). We first verified that all known exon 3 mutations could be detected in the heterozygous state by performing HRM analysis on DNA samples carrying known mutations (SN with c.150T>G/p.H50Q and lymphocytes with c.157G>A/p.A53T and c.136G>A/p.E46K), all of which were differentiated from control DNA (Fig. 2). As our aim was to detect somatic mutations at levels below 50%, the lowest proportion detectable was determined by HRM analysis of serial dilutions of genomic DNA carrying the H50Q and A53T mutations (for which adequate DNA was available) with wt DNA. HRM analysis against controls in triplicate revealed that mutant DNA proportions of 12.5% and 2.5%, respectively, were detectable (Fig. 3). All coding exons were amplified and analyzed by HRM in all 28 samples, but no shifts in melting curves signifying additional mutations were detected.

## Discussion

### DNA sources for Somatic Mutation Studies

**FIG. 4:**
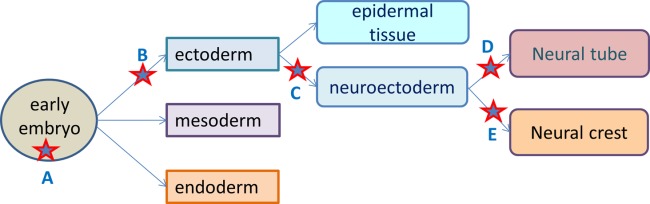
Schematic view of nervous system embryological derivation, with examples of somatic mutations affecting different cell populations (**A–E**). **A:** Mutation present in a proportion of cells from all germ layers. **B:** Mutation absent in nonectodermal tissues (eg, blood). **C:** Mutation present in all neuroectodermal tissue, including autonomic nervous system, but absent in skin. **D:** Mutation present throughout CNS. **E:** Mutation restricted to neural crest–derived structures (eg, adrenal medulla). As progenitor cell specification occurs very early, the diagram indicates the spatial distributions but not necessarily the precise timing of mutations.

We found no evidence of mosaicism for H50Q within the cerebellum and SN, but the possibility of a somatic mutation arising very early in embryogenesis and therefore being present in all brain cells, precluding detection of mosaicism within a single tissue, could not be excluded.^36^,^37^ Detection of this mutation in additional cases would help to prove an inherited rather than a somatic origin. While this article was under review, the same mutation was reported in a Canadian familial patient, further supporting the conclusion that this was an inherited variant.^38^ We analyzed samples derived from different brain regions using HRM, which has proven sensitivity for low-level somatic mutations that could be missed by Sanger sequencing, but did not find any to support our hypothesis. The possibility that somatic mutations were present in other regions could not be excluded. Careful consideration needs to be given to the choice of brain region for the detection of hypothesized somatic mutations, although this may be limited by availability, as in our case. The pathology of PD extends well beyond the mesencephalon-derived SN, and even regions affected early in its course are anatomically and embryologically distant (the olfactory bulb arising from the prosencephalon, the dorsal motor nucleus of the vagus from the rhombencephalon, and enteric neurons from the neural crest^39^). Although the orderly progression described by Braak^40^ may explain this, we propose that the widespread early distribution of PD pathology could be partly explained by somatic mutations arising by the third week of embryogenesis; this would lead to neurons carrying the mutations found in all 3 primary brain vesicles (prosencephalon, mesencephalon, rhombencephalon) and the neural crest (Fig. 4). In this case, use of any neuroectodermal tissue could allow detection of relevant somatic mutations, and brain regions in which neurons are resistant to the disease pathology (eg cerebellum in PD) would have the highest chance of detection of surviving neurons carrying these mutations. It is important to emphasize that, although it may seem counterintuitive, mutations occurring before the 3 germ layers split could lead to mosaicism not restricted to the ectoderm, as already demonstrated in the case of the Alzheimer's mosaic, with an 8% mutation proportion in the mesoderm-derived blood, which was initially not detected^23^; such very early somatic mutations could therefore be detected in blood if sufficiently sensitive methods were used.

### Pathology Variants, Mosaicism Pattern, and Spread

As variations from the Braak model are well established in a proportion of PD cases,^41^ we suggest that the precise pathology pattern in Lewy body diseases could depend on the distribution of neurons derived from the precursor that acquired the mutation. This is consistent with reports of apparently multicentric early SNCA pathology in some incidental Lewy body disease cases.^42^ The unexplained asymmetry in PD could relate to an asymmetric, stochastic somatic mutation burden. Lewy body diseases with distinct profiles, such as pure autonomic failure that is accompanied by hyposmia as severe as PD,^43^ would be a result of a mosaic pattern that differs from PD. In an analogous way, somatic mutations have been proposed as the cause of patient-specific variations in light-chain deposition in systemic light chain-amyloidosis.^44^ A continuum of risk of PD and age of onset would be dependent on the relative mutation load of an individual's neural tissue, as suggested for neurodegenerative disease in general.^12^ Inherited mutations would lead to the highest risk and earliest onset; whereas somatic mutations very early in neuroectodermal development could lead to a similar situation, those arising later and affecting fewer neurons would result in later-onset disease, and those with the least neuronal involvement could result in clinically silent incidental Lewy body disease. This is consistent with the observation that, in general, age of onset is earlier and severity greater in those who have inherited a *SNCA* mutation than in sporadic cases and would lead us to expect a higher prevalence of somatic mutations and higher mutation load in younger-onset cases. It is worth noting that many structures derived from the neural crest (which forms as the dorsal neural tube closes and whose derivatives include the sensory ganglia, sympathetic chain and preaortic ganglia, parasympathetic gastrointestinal ganglia, Schwann cells, adrenal medulla, and salivary glands) demonstrate SNCA pathology^45^; in the adrenal gland, pathology is seen in the medulla and related nerve bundles, but not in the mesoderm-derived cortex. Somatic mutations occurring late in the neural crest lineage could underlie the occasional reports of isolated Lewy body–like pathology in peripheral structures such as the adrenal medulla^46^ and in heart and stellate ganglia.^47^

Somatic mutations occurring later in neurodevelopment and therefore restricted to a small region could modify protein conformation locally, and this could still lead to spread by permissive templating^48^ and prion-like propagation of pathology.^49^ The concept of spread of neurodegenerative disease from a small focus of cells with a somatic mutation has already been proposed, with particular emphasis on amyotrophic lateral sclerosis.^12^,^50^ In this scenario, a negative result in brain-derived DNA would not exclude an initiating somatic mutation, which would be extremely difficult to detect, as neurons carrying it would be among the first to die after triggering the pathogenic process; a similar issue was discussed in relation to mtDNA mutations, with SN neurons with high mtDNA mutation levels likely to be lost early in the disease course.^34^ It is clear that harboring a *SNCA* mutation is not enough to lead to death of any type of neuron, as even in cases with inherited mutations, which all neurons carry, the distribution of pathology is very specific.^51^ Selective vulnerability of neurons in PD is determined by a number of properties,^52^ and the eventual pathological effect of a somatic mutation would depend on the biological effect of the mutation per se, the pattern of mosaicism and mutation load, selective neuronal vulnerability, and patterns of interneuronal propagation that may occur.

### Mosaicism for Other Types of Genomic Variants

We have focused our pilot work on attempting to detect small *SNCA* coding mutations, yet mosaicism for other types of genomic variation also merits consideration. CNV mosaicism is widespread,^53^ established very early in development,^54^ and present in normal brain.^55^ It is notable that a study of monozygotic twins discordant for PD or related phenotypes detected several copy number variations of somatic origin between cotwins of each pair, but as the DNA was derived from blood, no conclusion could be made on their pathogenicity.^56^ Somatic CNVs arise more frequently in chromosomal fragile sites,^57^,^58^ which include the regions where *SNCA*^59^ and *PARK2*^60^ reside. CNVs can be induced by DNA “replication stress,”^57^ which is likely in the rapidly dividing neuronal precursors.^61^ Mosaicism for aneuploidy (gains or losses of whole chromosomes) is very common in humans.^62^ Aneuploidy is common in neural progenitor cells, from which the cerebral cortex is derived, and a significant proportion appear to survive into adulthood as postmitotic neurons,^63^ with aneuploidy estimated in 10% of human brain cells.^64^ Aneuploidy for chromosome 21 is more common in Alzheimer's disease brains than in controls,^64^ and hyperploid neurons in general are selectively vulnerable.^65^ Finally, the LINE-1 retrotransposon constitutes around 20% of the human genome, and abundant LINE-1 somatic rearrangements by a “copy-and-paste” mechanism arising specifically in neuronal precursors in embryonic development already indicate significant acquired variability in normal human neuronal genomes.^66^,^67^ No data on relevance to disease are available, but occasional somatic LINE-1 insertions were reported in healthy control brains in both *SNCA* and *PARK2*,^66^ confirming that these genes are susceptible to such disruption.

### A Developmental and Evolutionary Perspective

Early somatic mutations leading to later neurodegeneration would be consistent with the “high initial load hypothesis” of the aging process, which proposes that initial damage of living organisms in fetal or early life may be responsible for later-onset degenerative disease.^68^ Even mtDNA mutations, generally considered to be acquired later in life, could arise in early development.^69^ Early somatic mutations may not, however, simply be accidents predisposing to later disease but could provide the basis for selection within an organism.^11^,^70^ This could be particularly relevant to selection of neuronal precursors and neurons that survive the massive apoptotic programmed cell death in early central nervous system development,^71^ which affects most neuronal populations, including dopaminergic neurons in the SN at the time of maximal competition for synaptic contact.^72^ A role for genetic variation (“chromosomal programming”) in the development of the nervous system, in a manner analogous to the immune system, was first suggested in 1967.^73^ Very recent data demonstrate that extreme aneuploidy is selected against in neurodevelopment.^74^ Multiple lines of evidence point to an important developmental role for SNCA, first in neuronal differentiation and later in synaptogenesis,^75^,^76^ with prominent perikaryal expression of SNCA in early development in the same neuronal groups later affected in PD.^77^ Therefore, we can speculate that certain somatic *SNCA* variants could be beneficial to neurons or their precursors in early development, undergoing positive selection within the organism, with PD a much later adverse consequence. As PD commonly develops past childbearing age, the mechanism that allows these early somatic changes would not be selected against and could indeed be favored by evolution if it allowed more genomic variation to provide a wider pool for selection of robust developing neurons. A similar explanation has already been put forward to account for LINE-1 mosaicism, suggesting that it favors generation of neuronal diversity, despite the possible risk of inducing neurological disease.^78^

## Conclusions

The investigation of the potential contribution of somatic mutation to sporadic neurodegenerative disorders is in its infancy. Further large-scale analysis of brain DNA is warranted to test the hypothesis that somatic mutations of *SNCA*, or indeed other genes, may contribute to sporadic PD. The HRM protocol we have developed could be used for detecting low-proportion somatic coding *SNCA* mutations, but although HRM sensitivity can be improved even further to 0.1%–1% by the use of a PCR modification that preferentially amplifies low-level mutants,^79^ the falling cost and increasing versatility of next-generation sequencing should also allow its use for detection of low-proportion somatic mutations by using very high depth of coverage for targeted genomic regions; this was very recently demonstrated for selected mitochondrial^80^ and nuclear^81^,^82^ genes. Additional techniques need to be considered for detection of larger variants, such as FISH for aneuploidy; custom CGH arrays can improve sensitivity for mosaic CNVs,^83^ but the improving detection of CNVs in next-generation sequencing data and droplet digital PCR sensitivity of 0.1%^53^ is likely to revolutionize CNV mosaicism studies. Ideally, multiple brain regions should be sampled, and other neuroectoderm-derived tissues could be analyzed. Demonstration of mosaicism for any mutations detected in brain would preferably be confirmed by analysis of other tissues, and their collection by research facilities should be considered; conversely, clinicians should bear in mind that the absence of a detectable mutation in blood cannot exclude a somatic mutation.

Although we have not detected any evidence of somatic mutations in our pilot work, we believe that we have provided strong grounds for considering a contribution of somatic mutations in *SNCA* or other genes to PD in at least a proportion of cases. If *SNCA* and other PD genes are particularly susceptible to somatic mutation and/or if certain deleterious somatic variants paradoxically confer a selective developmental advantage to neuronal precursors or neurons, somatic mutation could underlie a substantial proportion of PD and other Lewy body-type pathology.
