# Supplementary material for: Somatic Alpha-Synuclein Mutations in Parkinson's Disease: Hypothesis and Preliminary Data
Source: Mov Disord. 2013 May 14;28(6):705–12. doi: 10.1002/mds.25502 (PMC3739940; doi:10.1002/mds.25502)
Supplement: Supplementary file 1 [file mds0028-0705-SD1.docx]

| **DNA**  **source** | **Brain bank no** | **PM diagnosis** | **onset age** | **age at death** | **Duration**  **(years)** | **sex** | **presenting feature** | **later features** |  | **FH?** |
| --- | --- | --- | --- | --- | --- | --- | --- | --- | --- | --- |
| SN, cer | PA702/89 | iPD | 71 | 76 | 5 | m | L tr |  |  |  |
| SN, cer | PA446/90 | iPD | 50 | 66 | 16 | m | R tr, br, r | autonomic |  |  |
| SN, cer | PA673/88 | iPD, DLBD |  | 65 |  | m | bil r, br | dem, myoclonic jerks |  | Mother dem |
| SN, cer | P7/89* | iPD, CVD | 71 | 83 | 12 | f | R tr | dem, hal |  | |
| SN, cer | PA796/89 | iPD | 75 | 84 | 9 | m |  |  |  |  |
| cer | P2/90 | iPD, CVD | 66 | 77 | 11 | m | R br, r | dem |  |  |
| cer | P21/89 | iPD, AD | 62 | 87 | 25 | m | tr | dem |  | |
| cer | PA723/89 | iPD |  | 66 |  | m |  |  |  |  |
| cer | PA311/87 | iPD |  | 80 |  | m |  |  |  |  |
| cer | PA442/86 | iPD | 66 | 75 | 9 | m | bil r, br | dem |  | |
| cer | P46/86 | iPD |  | 74 |  | m |  |  |  |  |
| cer | PA266/90 | iPD, DLBD |  | 74 |  | f |  |  |  |  |
| cau | P29/92 | iPD | 51 | 62 | 11 | m | L br | dem, hal |  |  |
| cau | P32/92 | iPD | 78 | 82 | 4 | m | R tr |  |  |  |
| cau | P37 92 | iPD | 75 | 83 | 8 | f |  |  |  |  |
| cau | P38/92 | iPD, DLBD | 67 | 84 | 17 | f | L tr | dem |  | |
| cau | P40/92 | iPD | 55 | 77 | 22 | f | L tr, r, br |  |  |  |
| cau | P46/92 | iPD, CVD |  | 82 |  | m |  |  |  |  |
| cau | P11/92 | iPD | 60 | 78 | 18 |  | bil br, r |  |  |  |
| cau | P50/92 | iPD | 84 | 90 | 6 | f | R br, r |  |  |  |
| cau | P49/91 | iPD, AD | 58 | 72 | 14 | f | R tr | hal, dem |  | |
| cau | P57/91 | iPD | 60 | 71 | 11 | m | shuffling |  |  | Brother PD |
| cau | P50/91 | iPD | 78 | 83 | 5 | m |  | early falls, dem |  |  |
| cau | P3/93 | iPD | 54 | 72 | 18 | f | R tr | dem |  | |
| cau | P26/93 | iPD | 63 | 85 | 22 | f | R tr |  |  |  |
| cau | P42/93 | iPD | 72 | 79 | 7 | m |  |  |  |  |
| cau | P32/93 | iPD | 79 | 88 | 9 | f | R tr, r |  |  |  |
| cau | P22/94 | iPD |  | 75 |  | m |  |  |  |  |

**Supplementary Table S1: Details of patients whose brain DNA was analysed**.

The male to female ratio was 17:11, mean age at death 78 (SD +/- 7.3), mean age at onset where documented (n=21) 66.4 (SD +/- 9.8), mean disease duration at death for these 12 years (SD +/- 6.0).

* Case P7/89 was already reported to harbour the H50Q mutation (Proukakis et al, Neurology 2013;80:1062-1064).

Source= brain region from which DNA derived, SN= substantia nigra, cer= cerebellum, cau= caudate.

PM diagnosis: iPD= idiopathic Parkinson's disease, DLBD= diffuse Lewy body disease, AD= Alzheimer disease, CVD= cerebrovascular disease.

Presenting feature L=left, R=right, tr=tremor, bil= bilateral, br=bradykinesia, r=rigidity. dem= dementia, hal= hallucinations. FH= family history.

| name | Exon | Sequence | Product size | T_a_ used |
| --- | --- | --- | --- | --- |
| SNCA-1F | 1 | ccgagatagggacgaggagcac | 646 | 55 |
| SNCA-1R |  | AGACGCCCGTTTAGATCCACAGGT |  |  |
| SNCA-2F-ext | 2 | CCCCGAAAGTTCTCATTCAA | 235 | 51 |
| SNCA-2R-ext |  | CCCATCACTCATGAACAAGC |  |  |
| SNCA-3F-ext | 3 | GTATTGAAAACTAGCTAATCAGCA | 192 | 55 |
| SNCA-3R-ext |  | GCCACACTAATCACTAGATACTTT |  |  |
| SNCA-4F-ext | 4 | GTCACTGTTATTCTACCACCCTT | 249 | 55 |
| SNCA-4R-ext |  | GAACCGTAATCTCACCAGCTTA |  |  |
| SNCA-5F-ext | 5 | TTCCAAAACATGGTTCTGATT | 214 | 55 |
| SNCA-5R-ext |  | CAGGTTTTTGGTACTGTCTTGT |  |  |
| SNCA-6F-ext | 6 | GGAGCCATTTCCTATCTCATTG | 190 | 55 |
| SNCA-6R-ext |  | GCACTTGTACAGGATGGAACA |  |  |
| SNin2-F1* |  | tgttcagcacatgttaaagccttct | 553 | 55 |
| SNin2-Fc** |  | gttccttcttctagttttagga | 272 | 55 |

**Supplementary Table S2: Primers used for general PCR, sequencing and subcloning.**

The size of the product and T_a_ used for each exon primer pair are shown. *SNin2-F1 was used with 3R-ext in order to amplify exon 3 with 430 bp of intron 2. **SNin2-Fc was used with 3R-ext to generate a product containing rs72240586 and exon 3 for subcloning.

| Exon | Primer  direction | Sequence | T_a_ used | Amplicon  size | Number of melt domains |
| --- | --- | --- | --- | --- | --- |
| 2 | F | TCCAGTGTGGTGTAAAGGAAT | 65.2 | 187 | 1 |
|  | R | GAACAAGCACCAAACTGACAT |  |  |  |
| 3 | F | AGGCTAGCTTGAGACTTATGTC | 66.0 | 107 | 1 |
|  | R | TGGATATAAGCACAATGGAGC |  |  |  |
| 4 | F | CTACCACCCTTTAATCTGTTGT | 63.2 | 209 | 2 |
|  | R | TAACACAAAACGTACACAGCC |  |  |  |
| 5 | F | GCTACTTCATCATGTTCTTTTTGT | 68.0 | 160 | 2 |
|  | R | CTTGTTAGAAAGATTCAGCTTGG |  |  |  |
| 6 | F | ACAGTGTGTGCTGTCTTTTTGA | 63.2 | 97 | 1 |
|  | R | TCAAGAAACTGGGAGCAAAGA |  |  |  |

**Supplementary Table S3: primers and conditions used for HRM analysis.**
