# Supplementary material for: Somatic Alpha-Synuclein Mutations in Parkinson's Disease: Hypothesis and Preliminary Data
Source: Mov Disord. 2013 May 14;28(6):705–12. doi: 10.1002/mds.25502 (PMC3739940; doi:10.1002/mds.25502)
Supplement: Supplementary file 2 [file mds0028-0705-SD2.docx]

| Exon | Primer  direction | Sequence | T_a_ used | Amplicon  size | Number of melt domains |
| --- | --- | --- | --- | --- | --- |
| 2 | F | TCCAGTGTGGTGTAAAGGAAT | 65.2 | 187 | 1 |
|  | R | GAACAAGCACCAAACTGACAT |  |  |  |
| 3 | F | AGGCTAGCTTGAGACTTATGTC | 66.0 | 107 | 1 |
|  | R | TGGATATAAGCACAATGGAGC |  |  |  |
| 4 | F | CTACCACCCTTTAATCTGTTGT | 63.2 | 209 | 2 |
|  | R | TAACACAAAACGTACACAGCC |  |  |  |
| 5 | F | GCTACTTCATCATGTTCTTTTTGT | 68.0 | 160 | 2 |
|  | R | CTTGTTAGAAAGATTCAGCTTGG |  |  |  |
| 6 | F | ACAGTGTGTGCTGTCTTTTTGA | 63.2 | 97 | 1 |
|  | R | TCAAGAAACTGGGAGCAAAGA |  |  |  |

**Supplementary Table S2: primers and conditions used for HRM analysis.**

15 ng DNA was amplified for 40 cycles using novel primers with amplicons <210 bp. Optimisation for each exon was performed by varying annealing temperature and selecting the highest which did not lead to decline in fluorescence, and product size was confirmed by agarose gel electrophoresis. Additional primers GTTCCTTCTTCTAGTTTTAGGA (forward) and GCCACACTAATCACTAGATACTTT (reverse) were used for PCR (GoTaq, Promega), sequencing (UCL core facility), and subcloning (TOPO, Lifetech) of an amplicon including exon 3 and the rs72240586 intronic polymorphism. Exon and base numbering is based on ENSEMBL transcript ID 394986, protein ID 378437, with 6 exons and the initiating ATG in exon 2, with +1 corresponding to the A of the ATG initiating codon.
